# Low-grade B-cell non-Hodgkin lymphoma in the sternocleidomastoid muscle: a case report and review of literature

**DOI:** 10.3389/fonc.2026.1723488

**Published:** 2026-02-19

**Authors:** Guiping Peng, Xiang Wang, Miao Dai

**Affiliations:** 1 Department of Ultrasonic and Jiujiang City Key Laboratory of Cell Therapy, Jiujiang NO.1 People’s Hospital, Jiujiang, Jiangxi, China; 2 Department of Cardiovascular and Jiujiang City Key Laboratory of Cell Therapy, Jiujiang NO.1 People’s Hospital, Jiujiang, Jiangxi, China; 3 Department of Geriatrics and Jiujiang City Key Laboratory of Cell Therapy, Jiujiang NO.1 People’s Hospital, Jiujiang, Jiangxi, China; 4 Chronic Disease Management Center, Jiujiang NO.1 People’s Hospital, Jiujiang, Jiangxi, China

**Keywords:** extranodal marginal zone lymphoma (ENMZL), immunohistochemistry, intramuscular lymphoma, sternocleidomastoid muscle, watch-and-wait management

## Abstract

Primary non-Hodgkin lymphoma (NHL) arising in skeletal muscle is exceedingly rare. We present a case of low-grade B-cell NHL involving the sternocleidomastoid muscle. A 67-year-old male presented with an asymptomatic, slowly growing left neck mass. Imaging revealed a 17 mm × 11 mm intramuscular nodule, initially suggestive of a benign lesion. Excisional biopsy demonstrated lymphoid proliferation with muscle infiltration. Immunohistochemistry revealed CD20(+), PAX5(+), BCL2(+), and monoclonal IgH rearrangement, consistent with low-grade B-cell NHL, most likely extranodal marginal zone lymphoma (ENMZL), non-gastric/non-cutaneous type. Staging confirmed Lugano stage I disease. Given the indolent nature, the patient was managed with active surveillance. This case highlights an unusual intramuscular presentation of NHL, emphasizing the importance of histopathological and molecular evaluation for accurate diagnosis and tailored management.

## Introduction

Non-Hodgkin lymphomas (NHL) represent a heterogeneous group of lymphoid malignancies with diverse clinical, histological, and molecular characteristics. Among these, extranodal marginal zone lymphoma (ENMZL) of mucosa-associated lymphoid tissue (MALT) is a relatively common indolent subtype, accounting for approximately 7% of all NHL cases (1). ENMZL most frequently involves the stomach, salivary glands, thyroid, and ocular adnexa, but can arise in virtually any extranodal site (2). ENMZL must be distinguished from other small B-cell lymphomas, such as follicular lymphoma, chronic lymphocytic leukemia/small lymphocytic lymphoma (CLL/SLL), and mantle cell lymphoma, due to differences in prognosis and management (3). Extranodal involvement, particularly in the head and neck region, is uncommon, and primary intramuscular presentation is exceedingly rare, posing diagnostic challenges for clinicians and pathologists.

The diagnosis of ENMZL relies on a combination of histopathological examination, immunohistochemical profiling, and molecular studies to confirm clonality and exclude morphologic mimics. ENMZL cells exhibit an antigen profile similar to other MZLs, typically expressing CD19, CD20, CD79a, and CD22, while lacking CD5, CD10, CD23, and cyclin D1 (2, 4). However, phenotypic variations (e.g., CD5, IgD or CD10 expression in some cases, Bcl6 in large cells) should be noted for accurate diagnosis (5). However, due to overlapping features with reactive lymphoid hyperplasia and other small B-cell neoplasms, ancillary studies such as immunoglobulin heavy chain (IgH) gene rearrangement analysis are often necessary to establish a definitive diagnosis. Clinically, ENMZL often presents with localized disease at the involved extranodal site, and systemic symptoms such as fever, night sweats, or weight loss are uncommon (2). Given its indolent nature, some patients with asymptomatic, low-burden ENMZL may be managed with a watch-and-wait approach, particularly in elderly or comorbid individuals (6).

We present a rare case of a 67-year-old male with a primary intramuscular ENMZL manifesting as an asymptomatic neck mass, initially suspected to be a benign vascular lesion on imaging. This case highlights the diagnostic challenges associated with atypical presentations of low-grade lymphomas and underscores the importance of comprehensive histopathological and molecular evaluation in achieving an accurate diagnosis. Furthermore, it emphasizes the role of a multidisciplinary approach in determining optimal management strategies for early-stage ENMZL, particularly when considering conservative approaches such as active surveillance.

## Patients and methods

A 67-year-old male presented to our hospital with a palpable mass on the left side of his neck, first noticed approximately eight months prior. He denied systemic symptoms such as fever, night sweats, or weight loss. On the left side of the neck, lateral to the middle-upper segment of the sternocleidomastoid muscle, a quail egg-sized mass was palpable, firm in texture, with clear boundaries, no tenderness on palpation. No enlargement of other superficial lymph nodes was observed.

**Figure 1:**
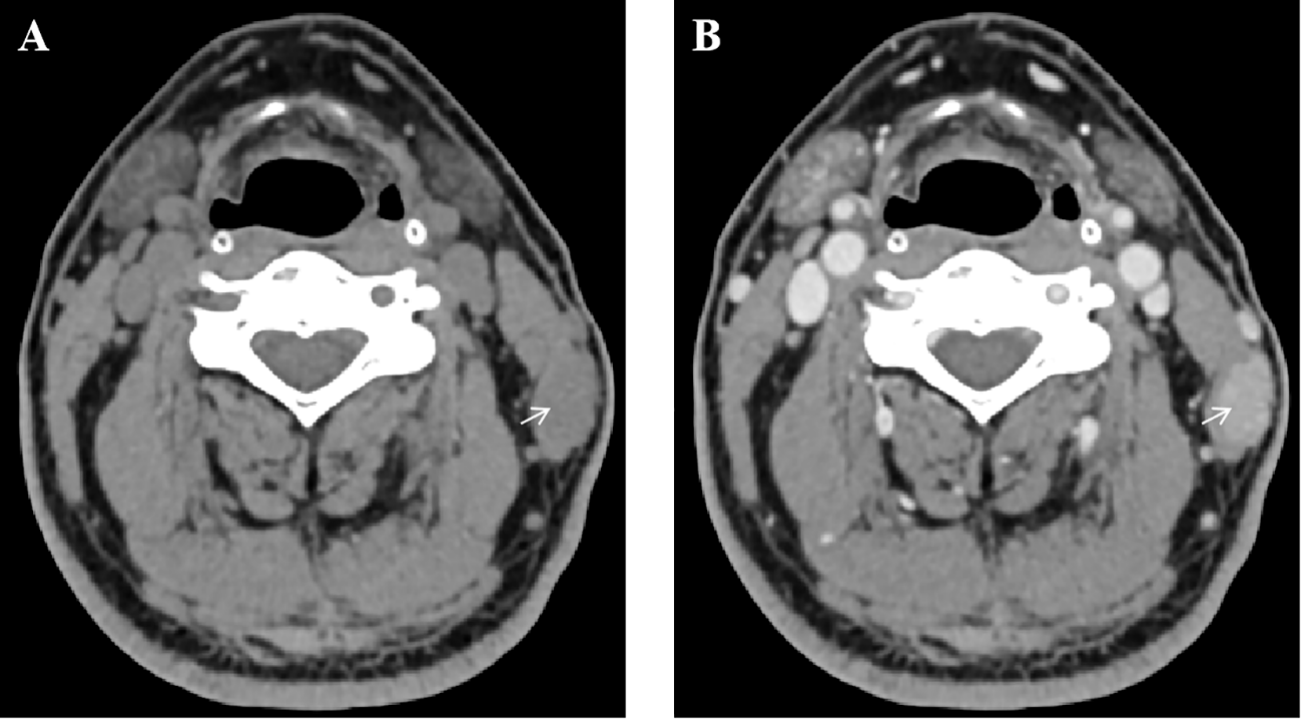
Contrast-enhanced neck CT scan demonstrating an intramuscular nodule. **(A)** Axial plain CT scan shows a hypodense nodule (arrow) within the left sternocleidomastoid muscle. **(B)** Axial contrast-enhanced CT scan in the same phase reveals progressive, uniform enhancement of the lesion (arrow).

Neck CT scan (plain and contrast-enhanced) revealed a 17 mm × 11 mm nodule within the left sternocleidomastoid muscle with unclear boundaries. The lesion exhibited progressive, uniform enhancement on contrast imaging, initially suggesting a benign etiology such as hemangioma (**Figure 1**). The white blood cell morphology was unremarkable. Chest and whole abdominal CT scan showed no significant abnormalities. Color Doppler ultrasound of superficial lymph nodes throughout the body revealed no significantly abnormal enlargement or echo of lymph nodes. Additionally, ultrasound of the thyroid and cervical lymph nodes, as well as electronic laryngoscopy, were performed and showed no evidence of lymphoma involvement in these regions. Dedicated imaging for lacrimal or salivary glands was not performed due to the absence of related clinical symptoms and the isolated presentation of the intramuscular mass.

**Figure 2:**
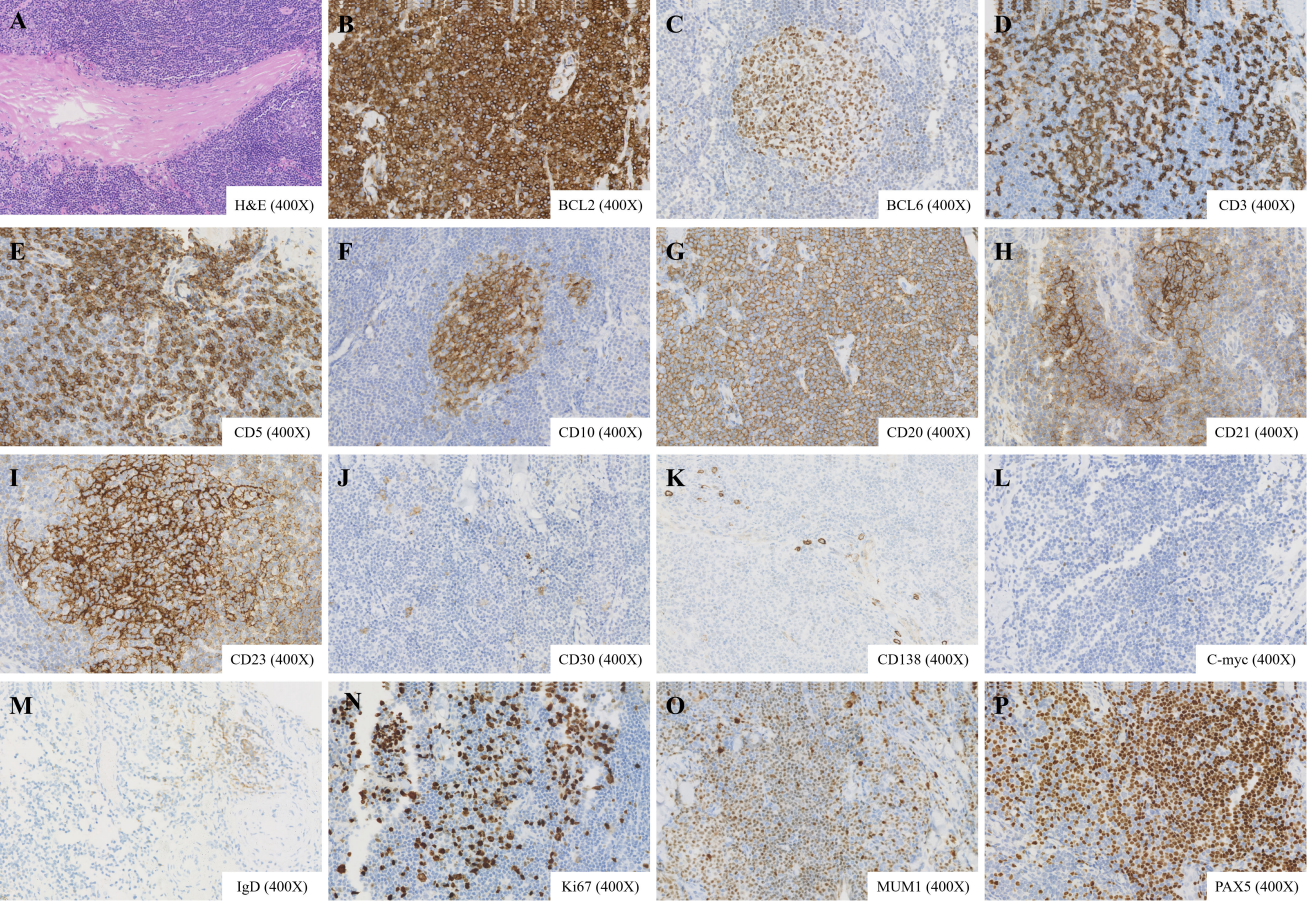
Histopathological and immunohistochemical features of the lymphoid nodule. **(A)** Hematoxylin and eosin staining reveals proliferative lymphoid tissue with reactive germinal centers, adenoid structures, and invasion into adjacent skeletal muscle (magnification ×400). **(B–P)** Immunohistochemical staining shows positivity for BCL2, BCL6, CD3, CD5, CD10, CD20, PAX5, CD21, CD23, CD30 (scattered), CD138 (partial), C-myc (scattered), IgD (scattered), Ki-67 (approximately 20%), MUM1 (partial), and PAX5 (magnification ×400).

The patient underwent excision of a left neck mass under general anesthesia. Gross examination of the specimen revealed a firm, well-circumscribed, gray-white tissue measuring 3.0 × 2.0 × 0.7 cm. Histopathological analysis of hematoxylin and eosin-stained sections demonstrated a dense, nodular lymphoid proliferation within the skeletal muscle. The lymphoid infiltrate was composed predominantly of small to medium-sized lymphocytes with irregular nuclear contours and scant to moderate pale cytoplasm, consistent with centrocyte-like or monocytoid B-cells. Focal areas showed infiltration and separation of individual skeletal muscle fibers, confirming intramuscular invasion. Within the lymphoid aggregates, occasional reactive germinal centers with tangible body macrophages were identified. Scattered epithelial structures, resembling residual glandular or ductal elements (adenoid structures), were also noted, possibly representing entrapped normal tissue or a component of the lesion’s microenvironment. No significant cytologic atypia, mitotic activity, or large transformed cells were observed. These morphologic features raised a strong suspicion of a low-grade B-cell lymphoproliferative disorder, prompting further immunohistochemical and molecular studies (**Figure 2A**).

Immunohistochemistry results were as follows: CD20 (+), PAX5 (+), CD3 (T cells +), CD5 (T cells +), CD21 (indicating FDC meshwork), CD23 (indicating FDC meshwork), Ki-67 (approximately20%+), CD10 (indicating germinal centers), BCL2 (+), CyclinD1 (-), SOX11 (-), BCL6 (indicating germinal centers), MUM1 (partial +), C-myc (scattered +), CD30 (scattered +), EBER (-), CK (-), IgD (scattered +), CD138 (partial +) (**Figures 2B–P**). IgH gene rearrangement analysis detected monoclonal rearrangement. Based on morphological findings, immunohistochemical staining, and the evaluation of B-cell clonality, the diagnosis is consistent with low-grade B-cell NHL, favoring ENMZL. Staging studies classified the lymphoma as Lugano stage I. A bone marrow biopsy was not performed given the localized nature of the disease, absence of systemic symptoms or laboratory abnormalities suggestive of disseminated disease, and the planned conservative management approach.

The patient has since undergone initial follow-up. At the 3-month post-excision evaluation, the patient’s complete blood count was within normal limits. Contrast-enhanced CT of the neck, chest, and abdomen showed significant reduction in the left sternocleidomastoid nodule, with no other abnormalities detected, consistent with a favorable response to local excision and supporting the current surveillance strategy.

## Discussion

This case presents an unusual instance of low-grade B-cell NHL localized within the SCM, a rare presentation that has seldom been reported in the literature. While ENMZL typically arises in mucosal-associated lymphoid tissue (MALT), involvement of skeletal muscle-particularly the SCM-is exceedingly uncommon. Our patient’s diagnosis was confirmed through histopathological and immunohistochemical analysis, revealing a clonal B-cell population consistent with ENMZL. This case expands the known anatomical spectrum of low-grade B-cell NHL and underscores the importance of considering lymphoma in the differential diagnosis of intramuscular masses, even in the absence of systemic symptoms or lymphadenopathy.

Primary lymphomas of skeletal muscle are rare (7), accounting for 0.1% to 1.4% of all extranodal lymphomas and 1.2–2.0% of all malignant muscle tumors (8), with most reported cases involving large muscle groups such as the thigh or upper arm (9, 10). A review of the literature reveals only a handful of documented cases of NHL involving the SCM, with the majority being diffuse large B-cell lymphomas (DLBCL) or high-grade variants (11). Low-grade B-cell lymphomas, particularly ENMZL (non-gastric, non-cutaneous type), presenting as an isolated intramuscular mass in this location, are exceptionally rare. To our knowledge, this is the first reported case of ENMZL confined to the SCM without concurrent lymph node involvement or disseminated disease.

The radiological features of this lesion initially suggested a benign vascular tumor, such as a hemangioma, due to its progressive contrast enhancement and well-circumscribed appearance. This highlights a diagnostic challenge, as intramuscular lymphomas often mimic benign soft tissue neoplasms on imaging. Previous studies have noted that MRI and CT findings in muscular lymphomas can be nonspecific, sometimes resembling sarcoma or myositis (9, 12, 13). Thus, histopathological evaluation remains the gold standard for definitive diagnosis.

The immunoprofile (CD20+, PAX5+, BCL2+, CyclinD1-, SOX11-) essentially excluded mantle cell lymphoma, including the rare CyclinD1-negative variant, and favored extranodal marginal zone lymphoma (ENMZL) over other small B-cell lymphomas. The presence of residual germinal centers and CD21/CD23-positive follicular dendritic cell meshworks is compatible with ENMZL. Low Ki-67 (~20%) and absence of c-MYC overexpression were consistent with an indolent course. Monoclonal IGH rearrangement confirmed neoplasia, and EBER negativity excluded EBV-driven lymphoproliferative disorders. Notably, the lack of EBV association (EBER-) ruled out EBV-driven lymphoproliferative disorders, which can sometimes present in extranodal sites.

Given the localized nature (Lugano stage I) and low-grade histology, our patient was managed with a watch-and-wait approach, consistent with current guidelines for early-stage indolent NHL (14). While surgical excision is not typically curative for systemic lymphoma, it may be sufficient for truly localized disease, as suggested in some case reports of primary muscular lymphomas. However, close follow-up is essential, as late relapses or progression can occur. Our surveillance plan includes periodic clinical evaluation every 3–6 months, along with cross-sectional imaging (CT or PET/CT) at 6- to 12-month intervals for the first 2–3 years, followed by annual imaging if the disease remains stable. Laboratory monitoring, including complete blood counts and lactate dehydrogenase, is performed at each visit to assess for systemic involvement or progression.

We acknowledge a limitation in our staging workup, as neither PET-CT nor bone marrow biopsy was performed. While these are standard procedures for comprehensive staging in lymphoma, the decision in this case was guided by the presentation of a solitary, small lesion with low-grade histology, the absence of clinical or laboratory findings suggestive of disseminated disease, and the planned conservative management strategy. This highlights a scenario where clinical judgment is applied within guideline frameworks. Nevertheless, the potential for occult disease, though considered low, necessitates our emphasis on vigilant long-term follow-up for this patient.

## Conclusions

This report describes a rare presentation of low-grade B-cell NHL confined to the SCM, expanding the clinical and anatomical spectrum of ENMZL. The case highlights the diagnostic challenges posed by intramuscular lymphomas, which can mimic benign lesions on imaging. A high index of suspicion, thorough histopathological evaluation, and molecular studies are crucial for accurate diagnosis. Further documentation of similar cases will help refine diagnostic and therapeutic approaches for this unusual manifestation of NHL.

## Data Availability

The original contributions presented in the study are included in the article/supplementary material. Further inquiries can be directed to the corresponding author.
